# Efficient utilization of date palm waste for the bioethanol production through *Saccharomyces cerevisiae* strain

**DOI:** 10.1002/fsn3.2175

**Published:** 2021-02-21

**Authors:** Arslan Ahmad, Summar A. Naqvi, Muhammad J. Jaskani, Muhammad Waseem, Ehsan Ali, Iqrar A. Khan, Muhammad Faisal Manzoor, Azhari Siddeeg, Rana Muhammad Aadil

**Affiliations:** ^1^ Institute of Horticultural Sciences University of Agriculture Faisalabad Faisalabad Pakistan; ^2^ Punjab Bioenergy Institute University of Agriculture Faisalabad Pakistan; ^3^ School of Food and Biological Engineering Jiangsu University Zhenjiang China; ^4^ Department of Food Engineering Faculty of Engineering University of Gezira Wad Medani Sudan; ^5^ National Institute of Food Science and Technology University of Agriculture Faisalabad Faisalabad Pakistan

**Keywords:** bioethanol, date palm waste, fermentation, *Saccharomyces cerevisiae*, yeast

## Abstract

Dates (*Phoenix dactylifera* L.) are rich in nutritional compounds, particularly in sugars. Sugars offer anaerobic fermentation, used for bioethanol production. Recently, researchers and industrialists finding ways to produce low‐cost bioethanol on large scale using agricultural wastes. Date palm residual is the largest agricultural waste in Pakistan, which can be the cheapest source for bioethanol production, whereas the current study was designed to explore the possible utilization and the potential of date palm waste for bioethanol production through *Saccharomyces cerevisiae* grown in yeast extract, Bacto peptone, and d‐glucose medium. The fermentation process resulted in the production of 15% (v/v) ethanol under the optimum condition of an incubation period of 72 hr and three sugars (glucose, fructose, and sucrose) were found in date waste. The functional group of ethanol (C_2_H_5_OH) was also found via Fourier‐transform infrared spectroscopy (FTIR) analysis. Therefore, *S. cerevisiae* could be recommended for ethanol production due to short fermentation time at 25% inoculum in 30°C and reduced the processing cost. Common date varieties of low market value are a preferred substrate for the process of producing industrial ethanol. Additionally, proximate analysis of date fruit by near‐infrared spectroscopy revealed moisture contents (16.84%), crude protein (0.3%), ash (9.8%), crude fat (2.6%), and neutral detergent fibers (13.4%). So, date fruit contains various nutrients for microbial growth for ethanol production.

## Graphical abstract

Ethanol produced from agricultural waste can be used for laboratory purposes, nutraceuticals, pharmaceuticals, and medicine. It can be used as a pure fuel or blended with gasoline used for vehicles.

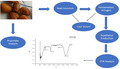

## 1 INTRODUCTION

Date palm (*Phoenix dactylifera* L.) belongs to the family Arecaceae. It is a tall, monocot, evergreen, dioecious plant (diploid) having 2n = 36. It is cultivated in most of the world's arid and semi‐arid areas. It is one of the oldest cultivated plants, which belongs to the Arabian Peninsula, and considered a vital character in daily life commodities for the people since 7,000 years (Sallon et al., 2020). The annual production of Pakistan in dates is 0.47 million tons, and it is the 6th leading producer of date palm in the world after Egypt, Iran, Algeria, Saudi Arabia, and Iraq (FAO, 2018). Though Pakistan is the major producer of dates in the world, a large quantity of fruits is wasted due to monsoon rains and low market value, resulting in significant economic loss to the farmers. There is a need for transforming the wastes of date palm into useful products for economic yield (Kumar et al., 2019). Boulal et al. (2016) used the date palm waste (DPW) to produce ethanol by using solar energy. The results revealed the production of ethanol (250 ml/kg) from DPW at 90°C. The waste recycling procedure of date fruit is supposed the foremost biological process inside the environmental structure that is aimed to have got the environmental balance. It is recognized that dates are the principal source of natural antioxidants which include carotenoids, phenolics, tocopherols, flavonoids, and ascorbic acid which may occur in different concentrations and forms according to the date palm genotype and postharvest processing mechanics. Similarly, date seed oil's total antioxidant activity depends on the type and different antioxidant concentrations, depending on date variety and method of extraction (Siddeeg, Faisal Manzoor, et al., 2019; Siddeeg, Zeng, et al., 2019; Younas et al., 2020).

Dates are usually complete food, biodegradable, and fermentable sugars offer anaerobic fermentation, used for bioethanol production and distillation afterward. Its sap is vastly nutritive due to high sugar content (60%–70%) and considers very good for microbial fermentation (Banaruee & Amirizad, 2017). If fermented molasses contains 16% sugar, it yields up to 5.6%–6% alcohol concentrations (Fadel et al., 2013). In fermentation processes, various microorganisms (*Zymomonas mobilis* and *Saccharomyces cerevisiae*) can be used to produce fructose and bioethanol. Generally, the use of mutants of these microorganisms decreases the consumption of fructose (Yang et al., 2016). There are three key classes of feedstocks that are used for bioethanol production such as lignocelluloses, starch, and sugars.

It is documented in the literature the effects of the sugar concentration, addition of different supplements to the molasses, pH, and some other factors affect very well the productivity of the ethanol (El‐Hussieny et al., 2020). Many microorganisms have been considered ethanologenic microbes. The yeast *S. cerevisiae*, *Kluyveromyces marxianus,* and *Z. mobilis* are better candidates for industrial alcohol production, and the efficiency was at maximum level with *S. cerevisiae* (88.19%) then *Z. mobilis* (81.59%) and *K. marixuanus* (70.34%) (Sadik & Halema, 2014). Production of ethanol from *S. cerevisiae* has higher efficiency and it grows very well in the presence of ethanol (13%), but the growing ethanol was completely forbidden at 14%. Because ethanol is recognized as an inhibitor of the growth of microbes, it has been testified that ethanol destructs the DNA in yeast cells and inactivate many enzymes (Tesfaw & Assefa, 2014). Bioethanol can be used as a crucial renewable and maintainable substitute hygienic fuel supply.

Ethanol derivative from waste productive has advantages as a substitute for fossil firewood which is harmless, renewable, eco‐friendly, and recyclable (Arshad et al., 2017). In recent times, in the gas sector, bioethanol manufacture surpasses 20 million gallons. Ethanol made from agricultural by‐products is renewable, and it produces bioethanol on a large scale which is beneficial over gas (Saini et al., 2015).

Governments also actively endorsed the commercialization, development, and identification of new technologies that were the substitutes of biofuels from the last three decades (Boulal et al., 2016). The process comprises the production of bioethanol, which captures the attention of numerous investors, consumers, and researchers for superior fuel sustainability. Therefore, the present study was aimed to investigate the efficiency of date palm fruit waste (DPFW) for bioethanol production and standardize the best combination of the substrate (DPFW) and inoculum (*S. cerevisiae)*.

## 2 MATERIALS AND METHODS

### 2.1 Sample collection and preparation

The sample of date palm cv. Dhakki was collected from Date Palm Research Substation, Jhang, Pakistan and brought to the Microbiology Laboratory of Punjab Bioenergy Institute (PBI), University of Agriculture Faisalabad, Pakistan for further use.

### 2.2 Proximate composition of date palm

Proximate composition was determined by near‐infrared spectroscopy (NIR). 250 g of fruit was taken to evaluate the moisture contents, ash, crude fat, starch, protein, and fiber through the NIR method. The fruit paste was completely dried and ground with the help of an electric grinder, and the powder was made. Then, the sample was kept in a tray and placed under NIR for evaluating the proximate composition (Bouhlali, Alem, Ennassir, Benlyas, Mbark, et al., 2017; Bouhlali, Alem, Ennassir, Benlyas, Nait, et al., 2017).

### 2.3 Ethanol production medium

Date palm fruit waste was used in the experiment to produce bioethanol. The fruit samples were washed and rinsed with water to remove any pebbles, sand, insects, or remaining plant parts. Then, pitting was done to separate seeds from the pulp, and grinding and dilution were made by adding distilled water in it and maintained pH 4 by adding 1 ml sulfuric acid (H_2_SO_4_). The microorganism *S. cerevisiae* was used in the fermentation process of the date because of its cheapness, productivity, and tolerance (El‐Hussieny et al., 2020).

### 2.4 Media preparation

Yeast extract, Bacto peptone, and d‐glucose (YPD) are used for the growth of *S. cerevisiae* and contains distilled water, bacto yeast extract, glucose, and bacto peptone. Media was prepared using Bacto yeast extract (10 g/L), Bacto peptone (20 g/L), and d‐glucose (20 g/L and *S. cerevisiae* (2 g/L).

Yeast extract (nutrimental source of yeast cell) (10 g) was added in 900 ml distilled water, and peptone (nitrogen source for yeast) (20 g) in 1 L of reagent bottle and neck was sealed with aluminum foil and cotton; then, the solution was autoclaved (121°C). Cooling of the solution was done after adding 100 ml glucose, which was sterilized, and inoculation was done in a laminar flow hood where germs were killed with the help of radioactive waves and prepared 99% ethanol. After the inoculation of YPD media, the solution was kept in a shaking incubator for 24 hr, 120 rpm at 30°C (Carrreira et al., 2011).

The shaking procedure of YPD media contributes to the yeast growth properly and after putting YPD media in the shaking incubator. Shaking was used to guarantee O_2_ supplement accessibility, air circulation and to keep a specific distance from the bacterial settlement on the bottle base which would bring out cell passing from the absence of supplement approachability. Moreover, shaking forestalls gives biofilm development, bacterial clusters development, and guaranteeing productive bacterial multiplication. Various concentration of inoculum was added into the date palm substrate, that is, 5%, 10%, 15%, 20%, 25%, 30%, 35%, 40%, 45%, and 50%. All the samples were kept in the incubator for 5 days at 30°C. The samples run in the rotary evaporator at 115 rpm at 78°C for 3–4 min and noted the value of each inoculum. All the samples were kept in the rotatory evaporator machine at 115 rpm and 78°C, and the reading was assured by an alcohol meter (Sadik & Halema, 2014).

### 2.5 Inoculum concentration

Inoculum concentration of *S. cerevisiae* was made by using YPD media and diluted to different volumes by making various concentrations of inoculum such as 5%, 10%, 15%, 20%, 25%, 30%, 35%, 40%, 45%, and 50% (Mojović et al., 2006).

### 2.6 Confirmation test of ethanol

The presence of ethanol was indicated by the change in the sample color when subjected to different chemical reagents. Alcoholmeter is used for the determination of the concentration of the alcohol in the given liquid at a specific temperature. The temperature at which the alcohol meter gave the best readings was 15–20°C. Alcohol which was prepared in the laboratory as well as distilled by rotatory evaporator gave a reading of 80% by alcohol meter (Tsukamoto, 1981).

### 2.7 Evaluation of functional group of ethanol

Evaluation of the functional group was determined by Fourier‐transform infrared spectroscopy (FTIR) MODEL (ALPHA) through the method used by León‐Martínez et al. (2011).

### 2.8 Estimation of total soluble sugars and reducing sugars

Anthrone reagent method as explained by Ahsen et al. (2019) was used to determine total soluble sugar content. Extract about 100 µl was taken, and 900 µl distilled water was added into it. One ml anthrone reagent was also added and feted for 8 min, then the mixture cooled at room temperature and absorbance was read at 630 nm using a microplate reader (BioTek). The amount of soluble sugar in the sample was calculated by using a standard graph prepared by plotting the concentration of the standard on the *x*‐axis versus absorbance on the *y*‐axis.

Reducing sugars were determined by following the method explained by Garriga et al. (2017) with some modifications. Ten g of 3,4‐dinitrosalicylic acid (DNS) was dissolved in 200 ml distilled water. Then, continuously and slowly stir and add NaOH solution in 150 ml H_2_O. Incubation of mixture held at 50°C with stirring slowly to get a pure solution and added 30 g of KNaC_4_H_4_O_6_.4H_2_O. In the end, the mixture was filtered with the help of filter paper and made the volume up to 1,000 ml by adding distilled water in it. The sample was stored at a temperature below 10°C in a dark glass bottle. A standard solution of glucose at the concentration ranged from 0.2 to 2 ml was prepared. One ml of 0.05 M NaOH buffer was added, and standard solutions were made in each test tubes and it was used as a blank. Then, 1 ml of samples was added in a suitable dilution to each test tubes by addition of 3 ml DNS reagent and 1 ml of 0.05 M NaOH and mix well. Tubes were incubated for 5 min, and then, absorbance was measured by spectrophotometer at 540 nm.

### 2.9 Statistical analysis

The data were subjected to a complete randomized design with three replications, and means were compared using the LSD test at a 5% level of significance.

## 3 RESULTS AND DISCUSSION

### 3.1 Proximate composition of date palm fruit

The results of the proximate analysis (Li et al., 2009) are presented in Table 1. The outcomes of proximate analysis of date palm fruit by NIR depicted that fruit is rich in proximate compounds. The results revealed that the number of moisture contents, crude protein, ash, crude fat, and neutral detergent fibers in dry matter was 16.8%, 0.3%, 9.8%, 2.6%, and 13.4%, respectively. The results of this study are in good arrangement with the previous research on the proximate analysis of different date palm varieties (Awan et al., 2018; Bouhlali, Alem, Ennassir, Benlyas, Mbark, et al., 2017; Bouhlali, Alem, Ennassir, Benlyas, Nait, et al., 2017). El‐Sohaimy and Hafez (2010) performed an analysis on date varieties from Egypt and found that these varieties contained crude fiber (5.20%), protein (3.00%), moisture (13.80%), fat (2.90%), and ash (2.13%). Ali et al. (2009) reported similar compositional characteristics of Omani dates. Dates fruit contains less fat and more fiber and higher ash content. The higher ash contents indicate the presence of higher mineral contents in date palm fruits (Awan et al., 2018). Our results and previous findings indicate that date palm is a good source of various nutrients, and these nutrients support microbial growth used in the fermentation process for ethanol production (Abd‐Alla & El‐Enany, 2012; Nazari, 2011).

**TABLE 1:** Proximate composition of date palm fruit by near‐infrared spectroscopy (NIR)

Proximate compounds	Dry matter (%)
Moisture	16.84
Protein	3.9
Neutral detergent fiber	13.4
Ash	9.8
Crude Fat	2.6
Crude fiber	6.47
Nitrogen free extract	70.95

### 3.2 Effect of inoculum percentage use on ethanol production

The production of ethanol (ml) in different inoculums used is given in Table 2. Ethanol production was zero when no inoculum (control) was used and significantly improved (*p* ≤.05) as the quantity of inoculum was increased from 5% to 25% for the media of *S. cerevisiae* (Table 2). On the other hand, when the inoculum amount was raised from 25% to 50% then the bioethanol production was significantly decreased (*p* ≤.05) by using the YPD media. In this study, inoculum concentration (25%) was observed as an appropriate concentration and the culture containing 25% of inoculum was used for ethanol production in further experimental studies. These conditions improved the process faster and significantly enhanced the production of ethanol. Appropriate temperature (30°C), pH (4.5), fermentation time (36–72 hr), sugar concentration (38 g/100 g), and inoculum size (25%) resulted in maximum ethanol yield (Azhar et al., 2017). This experiment predicted that the production of bioethanol from DPWs can be increased by using 25% inoculum concentration. The date palm fruit is rich in biodegradable sugar and provides bioethanol after fermentation for 72 hr at 30°C in the presence of yeast (Boulal et al., 2016). Many microorganisms (yeast) can be used to convert biomass to ethanol. *Saccharomyces cerevisiae* can produce 48.8 ml/kg ethanol from canned pineapple by‐products and 120.7 ml/kg ethanol from sorghum juice (Johansson et al., 2012). Mojović et al. (2006) also confirmed that using 10% inoculum of *S. cerevisiae* yeast in media, the production of ethyl alcohol was 4.25 ml/L (Table  2).

**TABLE 2:** Effect of inoculum percentage on ethanol production

Sr. #	Percentage of inoculum	Ethanol in ml from 100 ml
1	Control (no inoculum used)	1.04
2	5	2.16
3	10	1.8
4	15	4.5
5	20	7.42
6	25	11.4
7	30	7.68
8	35	7.67
9	40	7.2
10	45	5.8
11	50	1.65

**TABLE 3:** Infrared (IR) absorption frequencies of organic functional groups of different inoculum concentrations

Code	Concentration	Observed frequency	Reported	Functional group		Type of variation	Intensity	Reference
S1	Control	1,064.34	1,000–1,300	C‐O	Alcohol			Silverstein *et al*. (1981)
		3,349.67	3,200–3,600	O‐H	Alcohol	Stretch, H bound	Strong, broad	
S2	5%	1,064.516	1,000–1,300	C‐O	Alcohol			
		1,653.958	1,620–1,680	C = C	Alkene		Variable	
		3,272.72	3,200–3,600	O‐H	Alcohol	Stretch, H bound	Strong, broad	
S3	10%	1,066.715	1,000–1,300	C‐O	Alcohol		Variable	
		1,653.958	1,620–1,680	C = C	Alkene		Medium	
		3,283.72	3,200–3,600	O‐H	Alcohol	Stretch, H bound	Strong, broad	
S4	15%	1,066.715	1,000–1,300	C‐O	Alcohol		‐	
		1,653.958	1,620–1,680	C = C	Alkene		Variable	
		3,283.72	3,200–3,600	O‐H	Alcohol	Stretch, H bound	Strong, broad	
S5	20%	1,055.718	1,000–1,300	C‐O	Alcohol		Variable	
		1,645.161	1,620–1,680	C = C	Alkane		Medium	
		2,986.80	2,850–3,000	C‐H	Alkane		Strong	
		3,301.31	3,200–3,600	O‐H	Alcohol	Stretch, H bound	Strong, broad	
S6	25%	1,082.111	1,000–1,300	C‐O	Alcohol	Stretch	Strong	
		1,662.753	1,620–1,680	C = C	Alkene			
		2,969.201	2,850–3,000	C‐H	Alkane		Variable	
		3,347.501	3,200–3,600	O‐H	Alcohol		Medium	
S7	30%	1,064.51	1,000–1,300	C‐O	Alcohol	Stretch	Strong	
		1,646.161	1,620–1,680	C = C	Alkene			
		2,976.005	2,850–3,000	C‐H	Alkane		Variable	
		3,281.531	3,200–3,600	O‐H	Alcohol		Medium	
S8	35%	1,064.51	1,000–1,300	C‐O	Alcohol	Stretch	Strong	
		1,645.511	1,620–1,680	C = C	Alkene			
		2,969.208	2,850–3,000	C‐H	Alkane		Variable	
		3,310.11	3,200–3,600	O‐H	Alcohol		Medium	
S9	40%	1,064.51	1,000–1,300	C‐O	Alcohol	Stretch	Strong	
		1,653.95	1,620–1,680	C = C	Alkene			
		3,281.54	3,200–3,600	O‐H	Alcohol		Variable	
S10	45%	1,055.71	1,000–1,300	C‐O	Alcohol	Stretch	Strong	
		1,643.675	1,620–1,680	C = C	Alkene			
		2,978.23	2,850–3,000	C‐H	Alkane		Variable	
		3,327.146	3,200–3,600	O‐H	Alcohol		Medium	
S11	50%	1,073.311	1,000–1,300	C‐O	Alcohol	Stretch	Strong	
		1645.165	1,620–1,680	C = C	Alkene			
		3,283.52	3,200–3,600	O‐H	Alcohol		Variable	

### 3.3 Effect of temperature on ethanol yield

**FIGURE 1:**
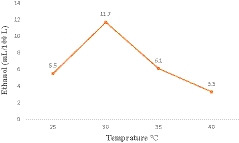
Effect of temperature on ethanol production

The effect of temperature on the production of ethanol is given in Figure 1. From Figure 1, it was observed that lower temperature resulted in a lower production rate of ethanol (5.5 ml), which could be possibly due to less activity of the yeast cell, because during fermentation if the temperature is low the activity of yeast cells decreases and results in lower ethanol production (Zabed et al., 2017). But the increase in the temperature had a significant (*p* ≤.05) effect on the ethanol concentration, the ethanol yield was maximum at 30°C. The ideal temperature for fermentation of molasses is reported between 28 to 32°C; hence, yield was maximum at 30°C (Azhar et al., 2017). The yield of the bioethanol was significantly decreased (*p* ≤ .05) to 6.1 and 3.3 ml when the temperature was increased to 35 and 40°C, respectively. Temperature is one of the most important factors that influence ethanol production (Lin et al., 2012). So, the production of ethanol was decreased with the increase in temperature (40°C), because the higher temperature may kill the majority of yeast cells and restricts the fermentation process (El‐Hussieny et al., 2020). Our results are also in similar to the findings of Fakruddin et al. (2013) who stated that *S. cerevisiae* and *S. unisporous* worked best and produced the maximum ethanol at 30°C.

### 3.4 Effect of sugars on ethanol production

**FIGURE 2:**
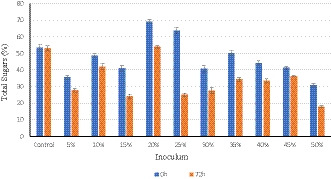
Overall comparison of total sugars consumed in all the inocula used

**FIGURE 3:**
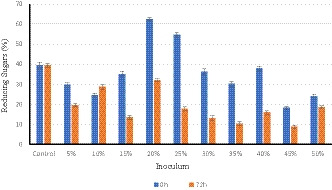
Overall comparison of reducing sugars consumed in all the inocula use

The efficiency of yeast to convert sugars into ethanol during the fermentation process is shown in Figures 2 and 3. As compared to control (without yeast inoculum), significant consumption (*p* ≤ .05) of sugars (total sugar and reducing sugars) by yeast was observed in all the inoculum used except The consumption of total sugar and reducing sugars was maximum (38.63 and 36.85 mg/100 g) at 25% inoculum of yeast, while there were nonsignificant differences (*p* ≤ .05) at the inoculum of 15%, 20%, 30%, and 35%. The results revealed that 25% inoculum of yeast consumed more sugars and produced maximum ethanol. The 2nd and 3rd best inoculum to produce ethanol was at 15% and 20% inoculum. The consumption of sugars was noticed for 120 hr but it was observed there was no consumption after 72 hr. The process of fermentation of sugars was active during the first 72 hr. After 72 hr, there was no consumption of sugars revealed by yeast. This may be due to the toxic effect of a longer fermentation period that is harmful to microbial growth (Zabed et al., 2014). However, the maximum yield of alcohol with the increase in sugar fermentation is justified from the findings of Bhatti et al. (2019) who stated that more sugar consumption increases the alcohol yield. In another study, Louhichi et al. (2013) found that fruits of three varieties of date palm produced the same amount of alcohol (25% V/V) after fermentation. The yield of alcohol from spoilage date fruits fermentation medium was greater as compared to synthetic medium containing the same sugar concentration (Abd‐Alla & El‐Enany, 2012). Our results are also similar to the results of Boulal et al. (2016) and Mehani et al. (2013) who have reported the fermentation time of sugars from 36 to 72 hr.

### 3.5 Fourier transformer Infrared Spectroscopy analysis of ethanol

**FIGURE 4:**
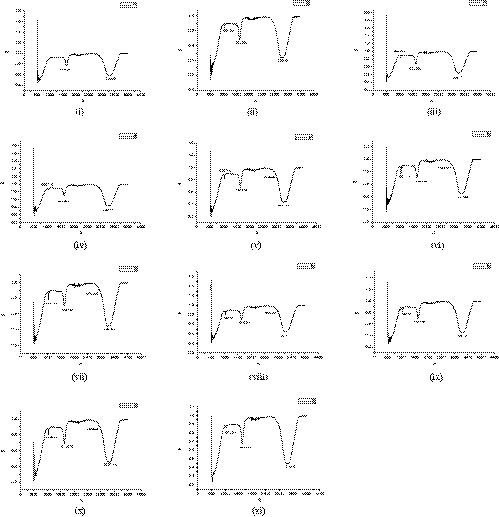
FT‐IR spectra of ethanol extract for different ratio of inoculum: (i) control, (ii) 5%, (iii) 10%, (iv) 15%, (v) 20%, (vi) 25%, (vii) 30%, (viii) 35%, (ix) 40%, (x) 45%, (xi) 50% A (wavenumber per cm) B (transmittance [%]

In Figure 4, FTIR analysis of ethanol produced confirms that the ethanol group (C_2_H_5_OH) is present. Because in FTIR, the region of 3,200–3,600 confirms the alcohol presence, which is shown in the analysis that a broad peak is present. Another peak is present in the region of 1,000–1,300 cm^−1^ which showed the CH_2_ group is also present. The FTIR spectra have recorded the range (400–4,000 cm^−1^) for more lucid FTIR graphs, and the full spectra are divided into five regions: 3,200–3,400, 1,620–1,680, 1,000–1,300, 2,850–3,000–500–700 cm^−1^. Two various spectral spheres in which one exposed CH elongating modes (2,850–3,000 cm^−1^) and the OH elongating (3,200–3,600 cm^−1^). And another covering the (C‐O) 1,000–1,300 cm^−1^, (C = C) 1,620–1,680 cm^−1^ vibration. The infrared spectra analysis also identified the vibration signs of biofuel from the waste date palm. The results revealed that wave numbers 2,900 and 3,300 cm^−1^ link to CH and OH molecule groups, respectively (Boulal et al., 2016; Manzoor et al., 2019, 2020; Mehani et al., 2013).

## 4 CONCLUSION

For efficient production of ethanol, the yeast *S*. *cerevisiae* was used for a better choice. It was found that 25% of the inoculum was the best treatment to use because its concentration was high and *S*. *cerevisiae* worked best at 30°C. The results depicted that the consumption of sugar was more from 36 to 72 hr, and after that, there was no consumption of sugars by yeast. The sugar consumption was more in 25% inoculum, and hence, there was high production of ethanol because more consumption of sugars results in more ethanol production. The results of FTIR analysis confirmed that the ethanol group is present which is C_2_H_5_OH. Proximate contents of date palm fruits by NIR revealed the diverse chemical composition with easily robot method.

## 5 Ethical Review

This study does not involve animal or human modeling.

## CONFLICT OF INTEREST

The authors declare that they have no conflict of interest.
